# Reducing Time to Analgesia for Sickle Cell Pain Episode Treatment in the Pediatric Emergency Department

**DOI:** 10.1097/pq9.0000000000000821

**Published:** 2025-06-04

**Authors:** Amelia F. Wong, Jaime K. Otillio, Abby K. Fahnestock, Christine M. Smith, Michael R. DeBaun, Emmanuel Volanakis, Lacey Noffsinger, Jeannie Byrd, S. Barron Frazier

**Affiliations:** From the * Division of Emergency Medicine, Department of Pediatrics, Vanderbilt University Medical Center, Nashville, Tenn.; † Department of Pediatrics, Vanderbilt University Medical Center, Nashville, Tenn.; ‡ Department of Pediatrics, Division of Hematology/Oncology, Vanderbilt University Medical Center, Nashville, Tenn.; § Vanderbilt-Meharry Center for Excellence in Sickle Disease, Nashville, Tenn.

## Abstract

**Introduction:**

Pain episodes are the most common emergency department (ED) presentation for patients with sickle cell disease (SCD). Prompt pain medication and frequent pain assessments are recommended. Our SMART aim was to reduce the time from ED rooming to first analgesia administration for children presenting with SCD pain from 50 to less than 30 minutes by June 2024.

**Methods:**

Children presenting to the ED with a diagnosis of SCD requiring opioids for pain were included. The primary outcome was time from rooming to analgesia. A key driver diagram, developed by a multidisciplinary team, informed our interventions and then implemented through plan-do-study-act cycles. Statistical process control charts were used to analyze data with Nelson rules to detect special cause variation. Secondary measures included frequency of pain assessments in the first 2 hours and ED length of stay.

**Results:**

From July 2020 to June 2024, there were 447 eligible encounters. Baseline data (n = 143) revealed an average time from ED rooming to analgesia of 50 minutes. Following interventions, including order set implementation, multidisciplinary collaboration, and incorporating the home action plan in the ED, special cause variation was detected with a centerline shift to 32 minutes. The median number of pain assessments in the first 2 hours of arrival improved from 2.2 to 2.7 with order set utilization. ED length of stay remained unchanged.

**Conclusions:**

Standardizing care with an order set increased the number of pain assessments. Incorporation of the SCD home pain action plan into the ED treatment pathway decreased the time to analgesia.

## Introduction

Sickle cell disease (SCD) is a common, inherited blood disease estimated to affect 104,000 to 138,900 individuals in the United States, primarily African Americans.^1^ A mutation in the β-globin gene leads to an abnormal sickling of hemoglobin. Pain episodes, previously referred to as sickle cell pain crises or vaso-occlusive crises, are caused by vaso-occlusion from sickled red blood cells.^2^ One-third of patients with SCD will have symptoms by a year of age, and most children with SCD are symptomatic by age 4.^3^ Pain episodes are the hallmark of SCD. Recurrent episodes significantly impact quality of life and lead to cumulative organ damage. Frequency of pain episodes, along with acute chest syndrome, is the most common predictor of death in patients with SCD.^4^ Pain episodes are the most common reason for hospital admission in patients with SCD, with an annual healthcare cost approximating $900 million.^5^ Reports estimate children with SCD miss an average of 10% of the school year due to pain episodes.^5^

Opioids are the mainstay of pain episode treatment. Consensus guidelines recommend rapid initiation of analgesic therapy within 30 minutes of triage or within 60 minutes of registration and to reassess pain and readminister opioids if necessary for continued severe pain every 15–30 minutes until pain is under control per patient report.^6^ Prior studies have demonstrated difficulty meeting consensus guidelines. African American race and the diagnosis of SCD both contribute to longer wait times when presenting to an emergency department (ED) compared with other groups with the same assigned triage level.^7^ Other barriers include ED census and acuity, staffing limitations, facility limitations, lack of guideline awareness among providers, and difficulty establishing timely intravenous (IV) access.^8^

We identified a need to reduce the time to analgesia and increase pain assessments for patients presenting with SCD pain episodes, given the significant delays and variability for this population to receive pain medication. Informed by the consensus guidelines, we aimed to reduce the time from ED rooming to analgesia for children presenting with SCD pain to less than 30 minutes by June 2024.

## METHODS

### Setting

This quality improvement (QI) initiative was conducted in a free-standing quaternary pediatric hospital ED that serves more than 65,000 patients annually. The ED is staffed by attending physicians, pediatric emergency medicine fellows, pediatric residents, emergency medicine residents, family medicine residents, and advanced practice providers. The hospital is home to an SCD center of excellence that emergency medicine providers consult for recommendations and needs for admission. The included population consisted of all patients who presented to our ED with a diagnosis of sickle disease with pain that required opioids and excluded patients who had opioids at another institution before transfer to our ED.

### Planning

The study was initiated in August 2021 in collaboration with a multidisciplinary team composed of ED physicians, hematology/oncology physicians, ED nurse, pediatric resident, and a pharmacist. An institutional review board determined that this QI project was exempt from human subject review.

**Fig. 1.**
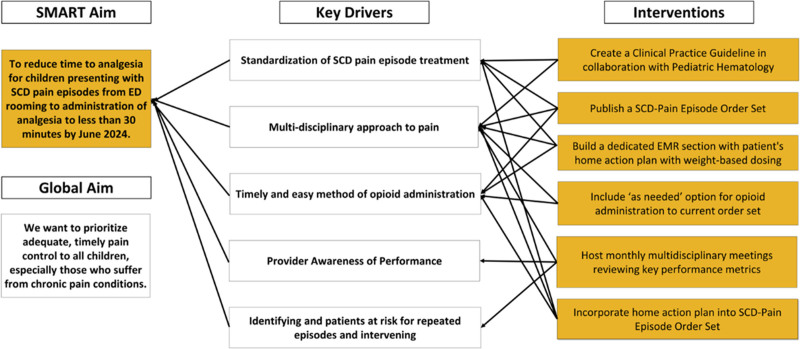
Key driver diagram.

Our multidisciplinary team developed a key driver diagram (Fig. 1). Retrospective baseline data were obtained from the electronic medical record (EMR) from July 2020 to August 2021, revealing a baseline average time from ED rooming to analgesia administration of 50 minutes with significant variability. The data were then followed prospectively and extracted from the EMR manually.

### Interventions

#### Order Set and Clinical Practice Guideline Introduced

**Fig. 2.**
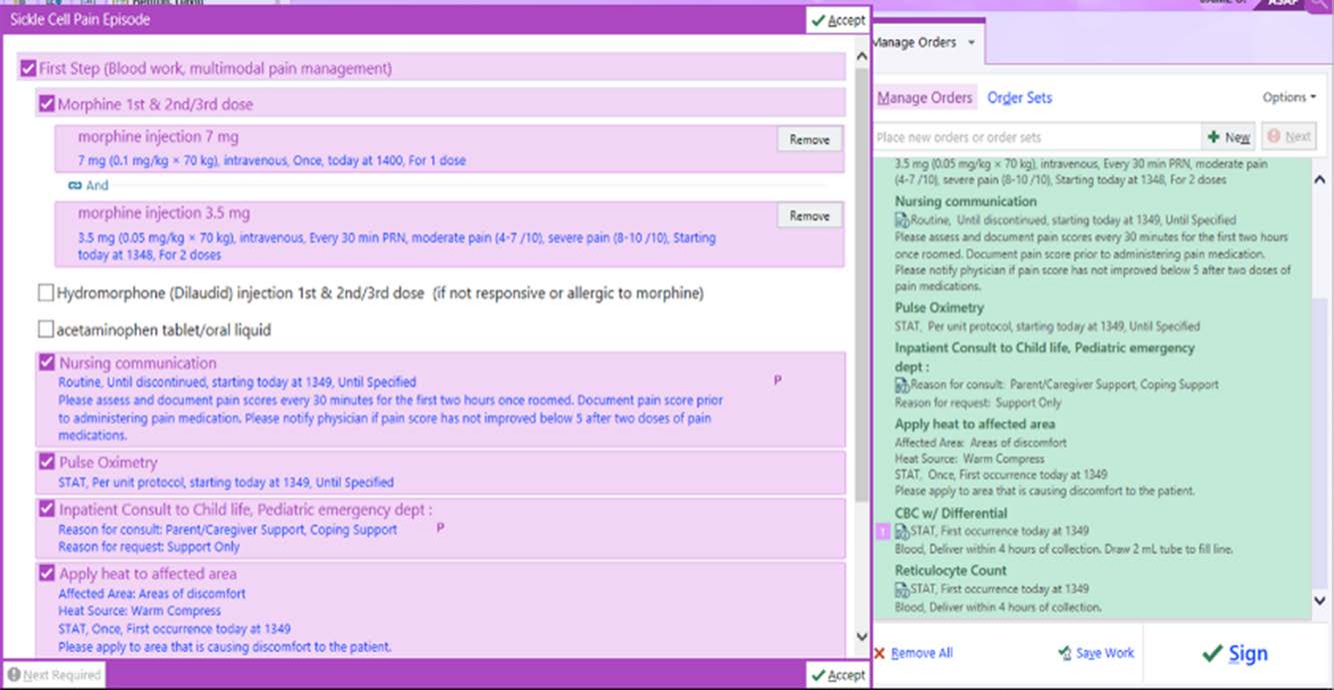
Initial sickle cell pain episode order set.

As part of the initial interventions in October 2021, a sickle cell pain episode order set (Fig. 2) was created concurrently with a clinical practice guideline. The order set encouraged providers to order three doses of morphine. The first dose was scheduled, and subsequent dosing was given as needed based on pain assessment and scores by nursing staff. The order set also included nonpharmacologic interventions for pain, including a heating pad and child life consult for distraction techniques. A complete blood count and reticulocyte count were included to help guide treatment.

#### Monthly Multidisciplinary Meetings

Having not yet seen substantial change in ED rooming to analgesia administration times, monthly multidisciplinary meetings began in June 2023. At these meetings, patients who presented with sickle cell pain episodes the month prior were reviewed with an ED provider, SCD clinic case management, hematology physicians, and nurses. Based on the review, provider feedback was provided if necessary. These meetings allowed for the identification of patients at risk for representation. Through these reviews, it was determined that most patients presenting to the ED had not taken their oral opioid as part of their home pain action plan before arrival.

#### Updated Home Action Plan in the ED EMR

**Fig. 3.**
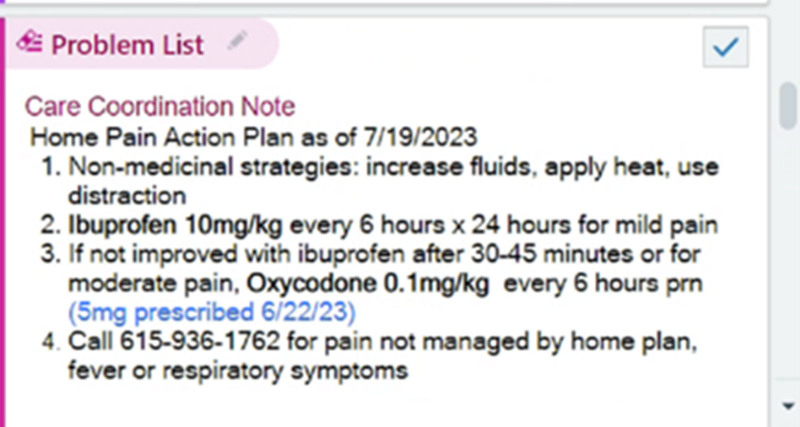
Home pain action plan in the “triage” tab of the ED EMR context.

To be consistent with the education provided in the outpatient sickle cell clinic and management done by the hematology team the home pain action plan needed to be readily available to the ED provider. In August 2023, the home pain action plan was added to the “Triage” tab of the ED context, and education on its location was disseminated at ED division meetings (Fig. 3).

#### New Order Set

To better align the practice of pain management between the sickle cell outpatient clinic and the ED, in December 2023, the order set was updated to encourage providers to order oral oxycodone if the patient had not yet received an oral opioid as part of their home pain action plan before arrival. Intravenous access was identified as a barrier to timely administration, and oral opioid administration aimed to mitigate this barrier when appropriate.

### Measures

The primary outcome measure was time from ED rooming to the first analgesia administration. Additional outcome measures included the median number of pain assessments, pain interventions in the first 2 hours, and the length of stay (LOS) in the ED. Our process measure was order set usage. By tracking the proportion of pain medicines ordered through the order set, we could evaluate adherence to the intended workflow to evaluate if our changes correlated to decreased time to analgesia. The balancing measure was the need for naloxone for opioid-induced respiratory depression. This balancing measure was selected to ensure efforts to expedite timely and appropriate analgesia did not inadvertently increase opioid-related adverse events, prioritizing patient safety.

### Analysis

QI charts (San Antonio, Tex.) were used to develop statistical process X-bar-S charts and run charts to examine measure changes over time. Nelson rules were utilized to detect special cause variation.

## Results

**Fig. 4.**
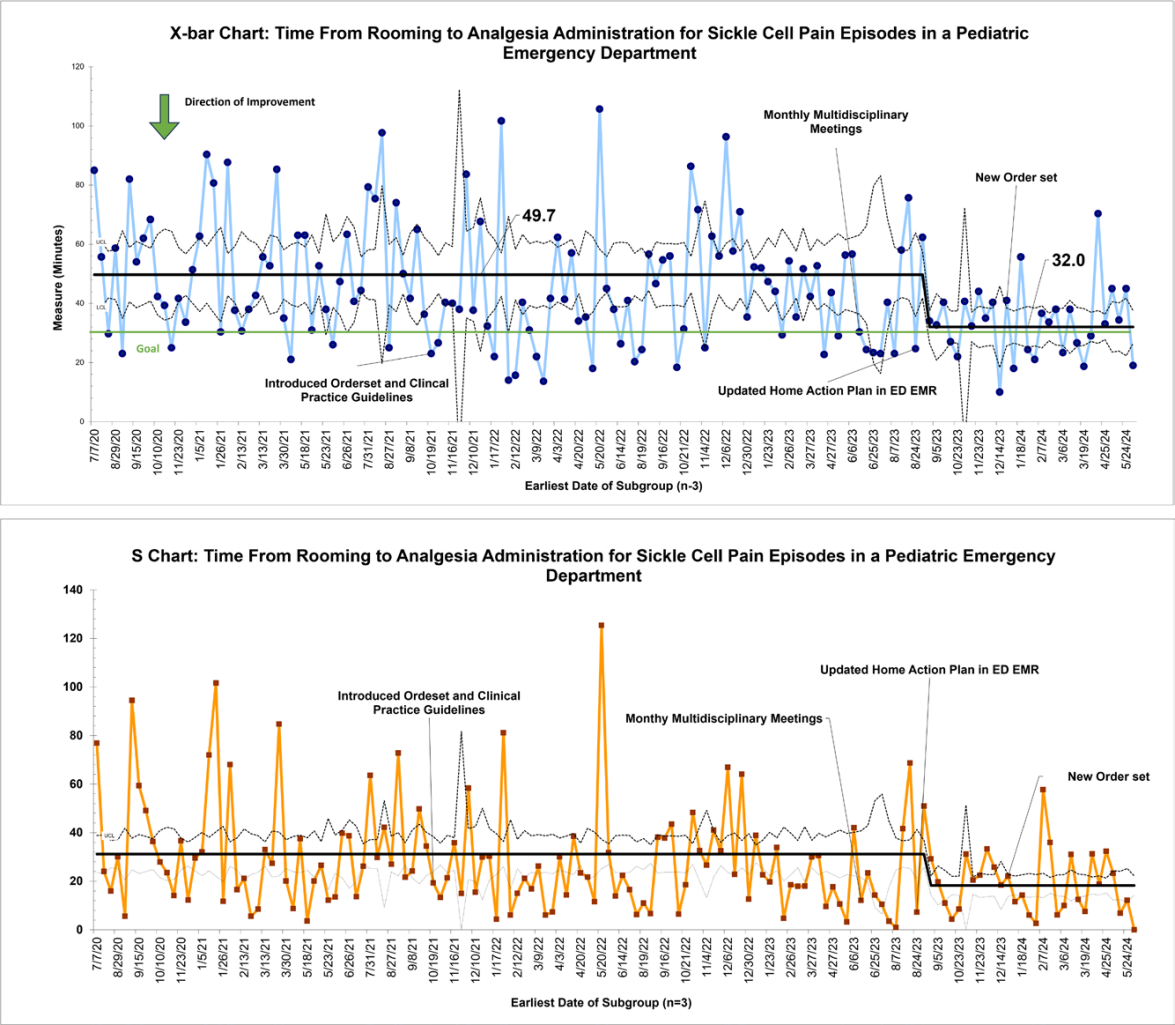
X-bar chart and S-chart for average time from ED rooming to analgesic administration.

There were 447 qualifying patient encounters from July 2020 to June 2024. Baseline data from 143 encounters revealed an average time from ED rooming to analgesia of 50 minutes with significant variability. In August 2023, following the integration of the home pain action plan into the ED pathway, special cause variation was detected with a centerline shift to 32 minutes (Fig. 4).

**Fig. 5.**
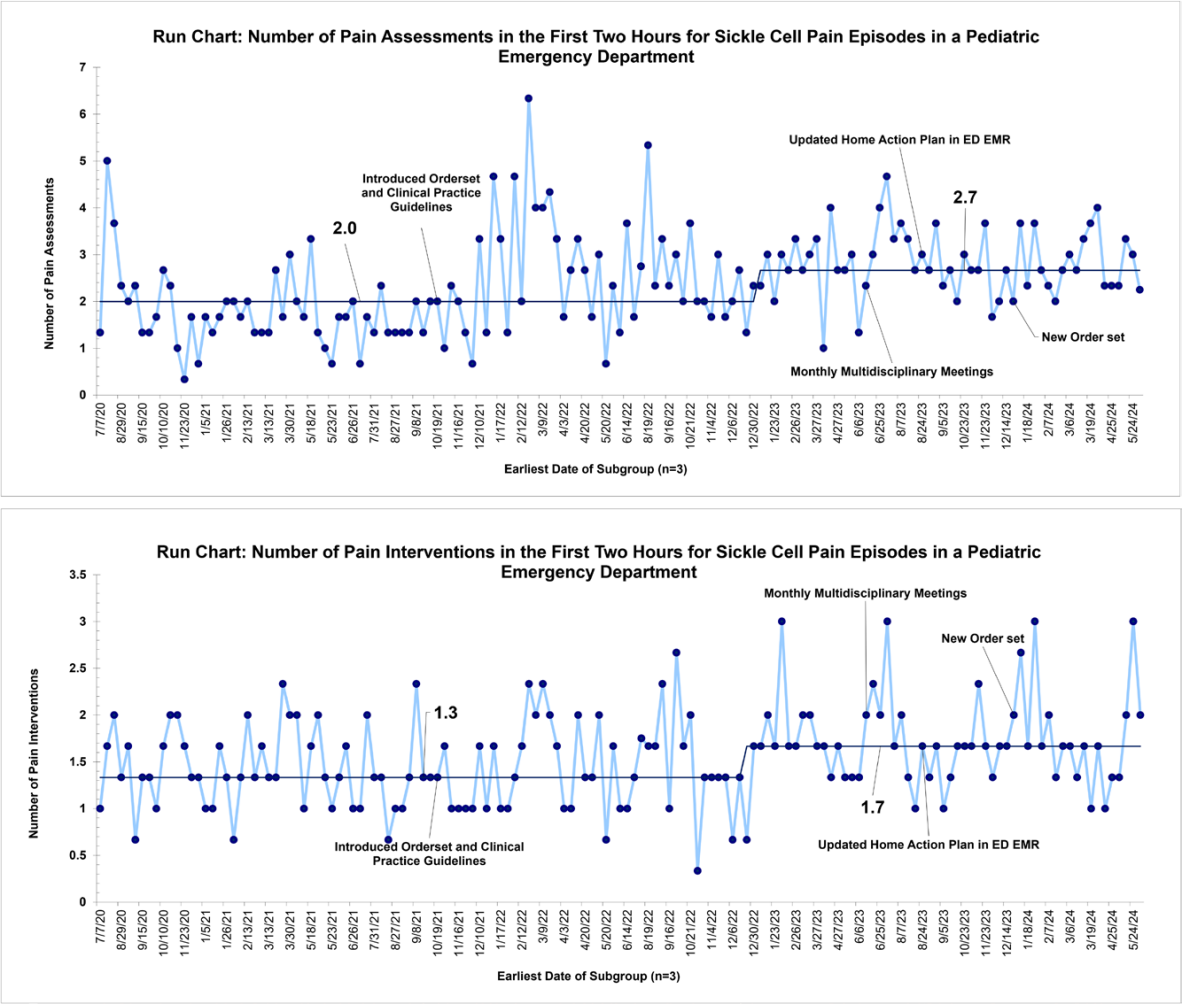
Run chart for median number of pain assessments and pain interventions in the first 2 hours in the pediatric.

**Fig. 6.**
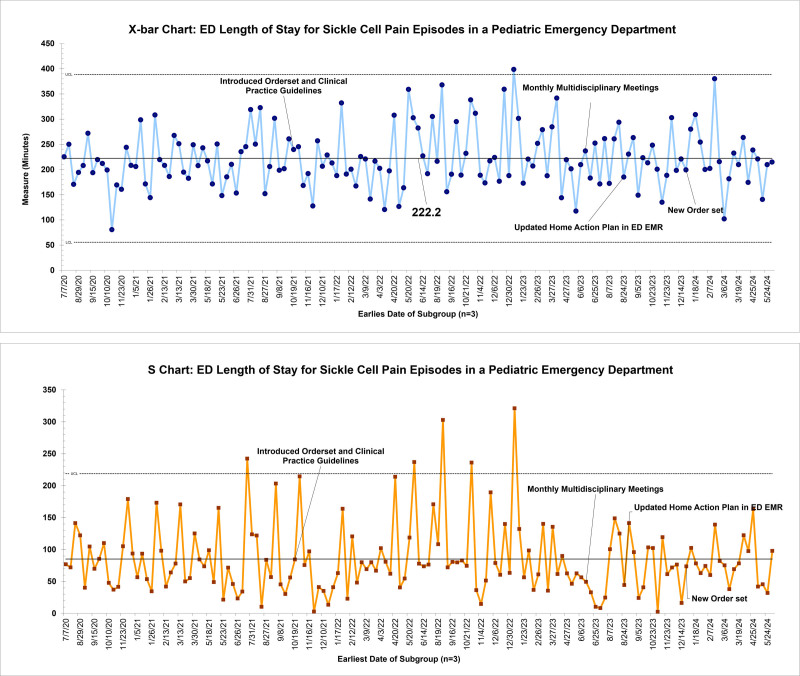
X-Bar chart and S-chart for average LOS.

The median number of pain assessments in the first 2 hours of arrival improved from 2.2 to 2.7 with order set utilization. The median number of pain interventions increased from 1.3 to 1.7 (Fig. 5). ED LOS remained unchanged at 222 minutes (Fig. 6). ED LOS for patients admitted for pain episodes remained 217 minutes. ED LOS for patients discharged from the ED remained at 233 minutes. There were no encounters requiring naloxone for opioid-induced respiratory depression.

## DISCUSSION

In this QI initiative, we reduced the average time from ED rooming to the first dose of analgesia from 50 to 32 minutes. Key interventions included creating and implementing a nursing-driven ED SCD pain episode order set, standing monthly multidisciplinary meetings for provider feedback and identifying patients at risk for recurrent sickle cell pain episodes, making the SCD home action plan easily accessible to ED providers, and incorporating the home action plan in the ED SCD pain episode order set.

The baseline data demonstrated that the initial average time to analgesia was 50 minutes, significantly exceeding the recommended guidelines. But notably, numerous special cause variations were noted within the baseline data, with many patients waiting for 200 minutes for pain medicines with variation in medications and doses used.

Prior studies have found that standardization of care with an EMR order set improved times to receive pain medicine.^9^ The development and introduction of a SCD pain episode order set were the first steps to standardize sickle cell pain management and address barriers in analgesia delay. By allowing nurses to administer opioids based on pain scores, the order set reduced reliance on provider availability, increasing the timeliness of pain management. The order set included a nursing order for pain assessment every 30 minutes for the first 2 hours. Order set utilization improved the number of pain assessments and pain medication administration in the first 2 hours.

Despite improvement in process measures, the initial SCD pain episode order set did not lead to the anticipated improvement in time from ED rooming to analgesia. This prompted the initiation of monthly multidisciplinary meetings with members from the ED and hematology team. All encounters from the prior month were reviewed and feedback was provided to ensure protocol adherence and patients at risk for representation were identified. Through these collaborative sessions, the multidisciplinary team identified a critical gap: many patients presented to the ED without having taken their home-prescribed oral opioids.

Recognizing this, the team took further steps to incorporate the home pain action plan directly into the ED’s workflow, ensuring that providers had immediate access to the patient’s individualized pain plan (Fig. 3).

Subsequently, the SCD pain episode order set was adjusted to incorporate the home action plan into the ED treatment pathway. This modification allowed for administration of weight-based oral oxycodone in the absence of taking their home oral opioid within the last 6 hours. This intervention not only incorporated a multidisciplinary approach to pain but also helped overcome the barrier of timely IV placement that other studies used IN Fentanyl to address.^10^ Our team chose to use oral opioids instead of IN Fentanyl to maintain consistency between the ED and outpatient management. Based on pharmacokinetics, oxycodone has a 10- to 30-minute onset of action lasting 3–6 hours, compared with IN Fentanyl with 7-minute onset of action and a 1-hour duration.^11,12^ Given the slight difference in onset of action and potential benefit of sustained pain control, we preferred oral fast-acting opioid medication. We believe this pivotal intervention led to special cause variation and a centerline shift from 50 to 32 minutes.

Although the time to analgesia improved, the overall ED LOS for patients with SCD pain episodes remained unchanged, even after segmenting our population between admitted and discharged dispositions. This finding suggests that other factors beyond pain management timing contribute to ED LOS.

Although further work is needed to meet the goal of pain medicines to be given within 30 minutes, our initiative offers a new method of integrating individualized home pain action plans directly into a standardized ED workflow. Our work builds upon previous treatment pathway initiatives by integrating outpatient and ED settings to promote care continuity.^8^Additionally, rather than relying on intranasal fentanyl to address IV access delays, we prioritized oral opioid administration, aligning with patients’ home regimens and longer therapeutic duration of pain control.^10^ This strategy not only simplified administration and improved timeliness but also ensured consistency in pain management across care environments. Embedding individualized care into a system-level protocol represents a scalable and sustainable model for improving outcomes in pediatric sickle cell pain management.

## LIMITATIONS

This QI initiative was conducted at a single pediatric quaternary care ED with an established center of excellence in SCD, which may limit generalizability to other institutions without similar infrastructure or resources. Data were manually extracted from the EMR, with variability in documentation introducing the possibility of transcription errors. Additionally, although the project tracked process and outcome measures over time, it did not assess patient-reported pain scores or satisfaction, which could provide a more comprehensive view of care quality.

## CONCLUDING SUMMARY

This QI initiative successfully reduced the time from ED rooming to analgesia for pediatric patients with SCD pain episodes by implementing a standardized order set and integrating individualized home pain action plans into the ED pathway.

Ongoing education and training for ED staff on the importance of prompt pain treatment and the utilization of the updated order sets will be crucial in sustaining these improvements. Additionally, this project has highlighted the need for improved transitions of care for patients admitted with SCD pain episodes from the ED to the hematology service, with ongoing initiatives to minimize gaps in pain medicines.

The reduction from 50 to 32 minutes from ED rooming to analgesia demonstrates the effectiveness of incorporating the home pain action plan in the ED pathway. Although the overall ED LOS was unchanged, the improvements in pain assessment, pain intervention frequency, and timely opioid administration reflect significant progress in the ED management of SCD pain episodes.

## ACKNOWLEDGMENTS

Kasey Jackson, MD, and Melanie Whitmore, PharmD, for their assistance in creating the Sickle Cell Pain Episode Order set.
